# Abnormal Development of the Teeth

**Published:** 1863-08

**Authors:** E. Ware Sylvester


### ABNORMAL DEVELOPMENT OF THE TEETH.

BY E. WARE SYLVESTER, D. D. S.

(Read before the American Dental Convention, Aug. 7th, 1863.)

When the architect designs to erect an edifice which shall not only be ornamental, but a lasting monument to his name, he is extremely careful to excavate deep and lay the foundation beyond all chances of disturbance, in order that the edifice in all coming time may remain unchanged and unchangeable. So the physiologist, if he would build a theory which shall stand the test of the most thorough scrutiny and investigation, must "begin at the beginning," and having so commenced, the causes of the disturbance in nature's laws can be often plainly discerned.

When God created man in his own image, we suppose him to have been perfect in his physical organization, and that Cain and Abel, at maturity, had well-formed dental arches, with full, round teeth inherited from their parents. In contrast with these mouths, let us take from one of our cities and from the class denominated the "upper ten" a lad of sixteen and mark the contrast. With an arch terminating almost in a point in front, the bicuspids on the opposite sides of the arch within about half an inch of each other, some of the upper incisor teeth falling within and some falling without the arch of the lower jaw, with an occasional space where a tooth ought to have been, and a half dozen nearly eaten up with gangrene, and we have the reverse of the picture in part.

True it is that some time has elapsed since Cain and Abel were perfecting their second dentition, but somewhere between that time and the present these changes have been produced, either suddenly or gradually. We are inclined to the gradual theory, although we by no means intend to countenance another theory of the gradual progression of the human species from the tadpole through their illustrious ancestors the monkeys. Nor has this deterioration been universal. Most of the Teutonic race as they land on our shores have well-developed dental arches and perfect teeth. The aborigines of our own country, where they have been uncontaminated with white vices and white cookery, have perfect teeth. Some years since I passed a day, with several assistants, in opening some of those vast mounds on the oyster coast of Georgia where the aborigines had been buried by thousands, and where the teeth, and often the alveolar process, were in a perfect state of preservation. In the examination of thousands of teeth I did not find a single one affected with caries, and but a single case of disease, and this specimen I now have in my collection, and this disease was apparently produced by wearing down the crown of the tooth until the pulp of a molar was injured, and this had produced suppuration at the apex of the fang.

We come then to the conclusion that the full dental arch and sound teeth are natural according to the designs of the Creator, and that a contracted arch and diseased teeth are the results of a series of violations of nature's laws.

We will notice then in the present paper five of these violations which have, in part, produced these disastrous results.

1. *Parental Influence.*—"Like begets like." "As is the father so is the son." These are truisms which have long since passed into proverbs, and are generally admitted. If both parents have well-developed mouths, and the child is reared in accordance with nature's laws, it will have similar masticators. If one of the parents has a defective dental apparatus and the other perfect, some of the children will inherit the "like" of the one, and sometimes of the other, and often the two mouths will seem to be blended in the offspring. And I suppose we have all seen cases like the following. The mother articulated the mouth in the usual manner, with the upper incisors closing in front of the under incisors; the father antagonizes perpendicularly in front.

Now it often occurs that the children of these parents will seem to have the right side of the mouth resembling the one parent and the left resembling the other. From the median line as a point, the two sides of the mouth are dissimilar, forming a difficult case to regulate and put in perfect shape.

Defective teeth on the part of one or both parents seem to follow the same law that "like produces like."

2. *Gestatory Influence.*—When a successful intercourse has been enjoyed between parents, and the womb impregnated, it would seem to be in accordance with the laws of our being that the husband and prospective father should forego his marital rights and privileges, and leave the prospective mother without future excitement, in order that she may perfect the embryo being committed to her charge. But we have every reason to believe that this is far from being the usual course, and that frequent sexual connection is indulged in, resulting in enfeebling the germ already commenced, and, as a natural consequence, preventing that full development which nature requires.

3. *Improper Diet.*—I now picture from the "upper ten." From the time that the infant first makes his voice heard to the age of maturity, there is almost one constant violation of nature's laws. Instead of allowing the mother's milk to cleanse the intestinal canal of the infant, he is immediately dosed with molasses and water, molasses and urine or some one of the thousand specifics which the cruel nurses have invented.

The mother, too enfeebled by previous sins, it may be, has no milk, and the aid of the cow and the confectioner are invoked to assist in rearing the dear little sickly infant, and the more unhealthy it is the more dear it is, as a matter of course. As soon as the dear little one can swallow, arrowroot and sago, jellies and cream, paregoric and assafetida, wines and custards, salts of tartar and quinine, candy and confections, are all made to play their part in producing an abnormal condition of both the first and second dentitions which are reposing quietly in their own proper places in the dental arch. This same course is pursued, and the nurse with money and little carriage is sent to the baker's and confectioner's to get something which the dear little one can eat, his appetite is so poor. The bread, too, if perchance he is so vulgar as to eat any, must be from the finest of the wheat, white and beautiful, with all the phosphate of lime or bone-producing material, already extracted from it and given to the dogs and horses. Do you wonder that such diet produces an abnormal development of the teeth?

4. *Impure Air.*—Most persons do not seem to realize that every adult person removes the oxygen from and nearly destroys one gallon of air per minute—a child more, in proportion to his size. Hence we find beautiful curtains around our beds, double sashes in our windows, cotton in all the crevices of the room, listing on the doors, and every contrivance imaginable to keep out the pure breath of heaven; with the air-tight stove, and the flaming gas burning up the air, and several persons in the room breathing it up, few ventilators are introduced, and the life-giving, teeth-forming pure air gets access to the infant and the youth only by stealth. Is it a wonder that gas-tar and nitrogen, the stench from feather beds and decaying horse hair, do not produce natural, healthy teeth.

5. *Want of Exercise.*—We are still in the region of Fifth Avenue. At first the delicate little infant is carried on a pillow by the attentive nurse in slippers, with soft and measured tread. In due time he is transferred, on downy pillows, to the beautiful baby carriage with steel springs, (the old cradle is an institution of the past, it shook the little one too much,) and on the soft Wilton carpet it is very gently drawn back and forth by its attentive nurse, and sometimes, at a year or more, it is drawn on the smooth flagging of the street, or carefully driven by Patrick in a curtained carriage, with walking horses, around the square, and when school days come, by the same conveyance it is carried to the very select school where plebeian feet or heaven's pure air seldom find admittance.

You tell me this picture is over-drawn. I have seen the picture, and it is true to the life; there are other similar pictures with less coloring, just as there are some children with good mouths, medium mouths, bad mouths, and very bad mouths.

In conclusion, we remark, give us two healthy parents with full dental arches and robust constitutions, let them obey with strict fidelity the laws of our physical being, let the infant be nursed, the youth fed and clothed, and aired in accordance with nature's laws, place him on the pony, (a live, trotting pony I mean,) put him in the garden with the trowel and the spade, let him get a little "*real estate*" on his hands, in his boots, (if he has any,) and even on his face. Put him in Dr. Lewis's training with light clubs and pure fresh air, and we will guarantee a dental arch which will compare favorably with that of Noah or Methuselah.

